# Characteristics of HIV-infected U.S. Army soldiers linked in molecular transmission clusters, 2001-2012

**DOI:** 10.1371/journal.pone.0182376

**Published:** 2017-07-31

**Authors:** Shilpa Hakre, Linda L. Jagodzinski, Ying Liu, Peter T. Pham, Gustavo H. Kijak, Sodsai Tovanabutra, Francine E. McCutchan, Stephanie L. Scoville, Steven B. Cersovsky, Nelson L. Michael, Paul T. Scott, Sheila A. Peel

**Affiliations:** 1 U.S. Military HIV Research Program, Walter Reed Army Institute of Research, Silver Spring, Maryland, United States of America; 2 Henry M. Jackson Foundation for the Advancement of Military Medicine, Bethesda, Maryland, United States of America; 3 U.S. Army Public Health Center, Aberdeen Proving Ground, Maryland, United States of America; Centers for Disease Control and Prevention, UNITED STATES

## Abstract

**Objective:**

Recent surveillance data suggests the United States (U.S.) Army HIV epidemic is concentrated among men who have sex with men. To identify potential targets for HIV prevention strategies, the relationship between demographic and clinical factors and membership within transmission clusters based on baseline *pol* sequences of HIV-infected Soldiers from 2001 through 2012 were analyzed.

**Methods:**

We conducted a retrospective analysis of baseline partial *pol* sequences, demographic and clinical characteristics available for all Soldiers in active service and newly-diagnosed with HIV-1 infection from January 1, 2001 through December 31, 2012. HIV-1 subtype designations and transmission clusters were identified from phylogenetic analysis of sequences. Univariate and multivariate logistic regression models were used to evaluate and adjust for the association between characteristics and cluster membership.

**Results:**

Among 518 of 995 HIV-infected Soldiers with available partial *pol* sequences, 29% were members of a transmission cluster. Assignment to a southern U.S. region at diagnosis and year of diagnosis were independently associated with cluster membership after adjustment for other significant characteristics (p<0.10) of age, race, year of diagnosis, region of duty assignment, sexually transmitted infections, last negative HIV test, antiretroviral therapy, and transmitted drug resistance. Subtyping of the *pol* fragment indicated HIV-1 subtype B infection predominated (94%) among HIV-infected Soldiers.

**Conclusion:**

These findings identify areas to explore as HIV prevention targets in the U.S. Army. An increased frequency of current force testing may be justified, especially among Soldiers assigned to duty in installations with high local HIV prevalence such as southern U.S. states.

## Introduction

The United States (U.S.) national HIV/AIDS strategy (NHAS) was initiated in 2010 for a coordinated national response to the HIV epidemic in the U.S. The 2015 update focused on increased HIV testing, access to, and engagement in, medical care, viral suppression, and access to HIV pre-exposure prophylaxis.[1] The Centers for Disease Control and Prevention (CDC) developed the National HIV Surveillance System (NHSS) to monitor and characterize the U.S. HIV epidemic.[2] Findings from analysis of the molecular component of NHSS surveillance data identified priority areas aligned with the NHAS goal for HIV prevention programs to reduce new infections. Evaluation of NHSS 2001–2012 HIV-1 *pol* sequence data revealed that of 32% who had genetically similar HIV, significantly more HIV-infected men (35%) were in transmission clusters than women (19%). Among men who reported having sex with men (MSM), 92% were linked to other MSM (including MSM who were injecting drug users); MSM, especially older black MSM, were commonly linked to heterosexual women.[3]

New U.S. Army surveillance data suggest the HIV epidemic is concentrated among MSM.[4] A recent investigation, which included sequence and interview data, among HIV-infected Soldiers located at a single Army installation established that a majority of HIV transmission networks within an eighteen-month time period were among MSM.[5] We sought to evaluate factors associated with membership within a transmission cluster Army-wide and over a longer time period in an effort to identify characteristics of Soldiers who would benefit most from HIV prevention services.

## Methods

### Study population

The study population consisted of all Soldiers in active service and newly-diagnosed with HIV-1 infection from January 1, 2001 through December 31, 2012. A single available baseline partial *pol* sequence, generated for routine HIV medical care for each HIV-infected Soldier, was analyzed for evidence of transmission clusters.

### Ethical considerations

The U.S. Army Public Health Center’s Public Health Review Board (#146–12) and the Walter Reed Army Institute of Research Institutional Review Board (#1861-C) determined HIV surveillance program activities were public health practice.

### HIV diagnosis

HIV infection has been identified among Soldiers with standard U.S. diagnostic algorithms in use since 1989. Enzyme-linked immunoassay (EIA) initial screen-reactive specimens were tested in duplicate with the same EIA assay. Repeat reactive samples were reflexed to a confirmatory HIV-1 Western Blot assay (Genetic Systems HIV-1 Western Blot, Bio-Rad Laboratories, Redmond, Washington, U.S.). Enhancements to the Army algorithm in 2009 included replacement of a second generation EIA assay with a third generation EIA assay as well as incorporation of a qualitative HIV-1 ribonucleic acid assay to detect acute and/or primary HIV infection; the enhanced algorithm reduced the diagnostic window to 14–18 days post-infection.[6]

### Demographic and clinical data

The Army HIV surveillance program, enhanced in 2012,[5] utilizes prospectively collected risk behavior data, existing longitudinal data from medical surveillance (Defense Medical Surveillance System, Armed Forces Health Surveillance Branch) and laboratory databases, and data generated in the course of routine clinical care such as sequence data from antiretroviral resistance testing, baseline CD4 counts, HIV viral load, and co-infection with the sexually transmitted infections (STIs) hepatitis B (HBV) and C virus (HCV), syphilis, *Neisseria gonorrhoeae* (NG), and *Chlamydia trachomatis* (CT).[7]

### Sequence data

A 918 base pair partial *pol* sequence available for any Soldier diagnosed with HIV-1 infection from 2001 through 2012 was compiled and aligned using Geneious Pro 5.6.7.[8] Sequence alignment was further refined manually. HIV-1 subtype designations and transmission clusters, defined as ≥2 linked sequences, were identified from phylogenetic analysis of sequences using the maximum likelihood (ML) method and Kimura-2-parameter (K2P) model in MEGA6 (S1 Fig).[8, 9] Forty references consisting of pure HIV-1 subtype and main circulating recombinant forms (CRFs) were used to identify and designate HIV-1 subtypes (S1 Table). Recombinant subtype analysis was performed using a combination of HIV-1 Genotyping Tool[10], Jumping Profile Hidden Markov model [11], visual inspection, and construction of subregion ML trees with bootstrap analyses to confirm breakpoint assignments. Bootstrap values of 95 percent or higher and a genetic distance cutoff value of ≤1.5% (≤0.015 nucleotide substitutions per site) were used to identify sequences that were highly related to each other (i.e. a cluster). The cutoff value was established by identifying an optimal intra-cluster genetic distance using available sequences for all Soldiers (S2 Fig). The Tamura-Nei 93 distance model was used as well to confirm sequence relatedness and showed no significant differences from the K2P model (S3 Fig). Transmitted drug resistance mutations were identified using the World Health Organization’s 2009 standard surveillance list available from the Stanford HIV database; any resistance to non-/nucleoside reverse transcriptase or protease inhibitor resistance was considered.

### Analysis

Demographics (age, self-reported race, sex, occupation, pay grade, service rank) and other characteristics (location of assignment, STI co-infection, HIV specialty care: CD3/CD4, viral load, antiretroviral resistance testing, antiretroviral treatment) at/proximal to diagnosis were evaluated for all HIV-infected Soldiers and compared for those who were linked in transmission clusters to those who were not. Only transmission clusters for the study population (Soldiers in active service only and not the reserve component) were selected for further univariate and multivariate analysis; sequence data, demographic and other characteristics of interest were either not available or incomplete for HIV-infected Soldiers who were not in active service. Univariate and multivariate logistic regression models were used to evaluate, and adjust for, the association between characteristics and cluster membership. In general, sub-categories having lower risk (or normative sub-categories) were chosen as reference levels in multivariate analysis. Data were managed and analyzed using Statistical Analysis Software version 9.3 (SAS Cary, North Carolina).

## Results

### Study population

A total of 995 Soldiers in active service were newly-diagnosed with HIV-1 from January 1, 2001 through December 31, 2012. A majority (97%) were male, of non-white race (74%), with a median age of 27.0 years (interquartile range (IQR) 23.0–35.0) (Table 1). At diagnosis, 64% were assigned to a duty station in the southern U.S., 37% had a seronegative HIV test within the past year (median 437.0 days, IQR 258.0–702.5 days), and 52% had been in service for 5 years or less (median 5.0, IQR 2.0–11.0). Ninety percent (n = 896) of HIV-infected Soldiers had evidence of HIV specialty care within a median 35 days (IQR 18.0–58.0) of diagnosis. Almost half (43%) of HIV-infected Soldiers had a viral load of 10,000 copies/ml or more (median 21503.5, IQR 4888.0–69317.0; CD3/CD4 (cells/μl) median 445 (IQR 330.0–593.0)) (Table 1).

**Table 1:** Demographic, service-related, and clinical characteristics of 995 HIV-infected Soldiers newly diagnosed from 2001–2012, overall, and by transmission clustering.

Characteristic at Diagnosis	Value	All Cases n = 995		Clustered n = 151		Not Clustered n = 367	
Age (years)	18–20	71	(7)	14	(9)	20	(5)
	21–24	275	(28)	47	(31)	100	(27)
	25–29	254	(25)	43	(28)	107	(29)
	30–34	140	(14)	18	(12)	55	(15)
	35–39	111	(11)	18	(12)	36	(10)
	40+	144	(14)	11	(7)	49	(13)
Race, ethnicity	White, non-Hispanic	260	(26)	27	(18)	98	(27)
	Black, non-Hispanic	583	(59)	103	(68)	210	(57)
	Other	152	(15)	21	(14)	59	(16)
Sex	Male	964	(97)	148	(98)	359	(98)
	Female	31	(3)	3	(2)	8	(2)
Marital status	Single	563	(57)	85	(56)	215	(59)
	Married	369	(37)	59	(39)	130	(35)
	Other	52	(5)	7	(5)	18	(5)
Highest education attained	High school or less	744	(75)	121	(80)	272	(74)
	Some college or more	212	(21)	28	(18)	83	(23)
Length of service	1–2	259	(26)	40	(26)	98	(27)
	3–5	259	(26)	40	(26)	87	(24)
	6–8	142	(14)	28	(18)	65	(18)
	9+	331	(33)	43	(28)	117	(32)
Pay grade	E1-E4	497	(50)	72	(48)	173	(47)
	E5-E9	386	(39)	68	(45)	148	(40)
	Officer	104	(10)	11	(7)	43	(12)
Occupation category	Combat	147	(15)	31	(20)	52	(14)
	Health Care	131	(13)	15	(10)	51	(14)
	Other	717	(72)	105	(69)	264	(72)
Calendar year	2001	67	(7)	1	(1)	10	(3)
	2002	68	(7)	0	(0)	12	(3)
	2003	78	(8)	1	(1)	15	(4)
	2004	64	(6)	2	(1)	18	(5)
	2005	71	(7)	5	(3)	16	(4)
	2006	70	(7)	12	(8)	15	(4)
	2007	70	(7)	15	(10)	31	(8)
	2008	103	(10)	22	(15)	52	(14)
	2009	93	(9)	12	(8)	47	(13)
	2010	95	(10)	20	(13)	36	(10)
	2011	105	(11)	23	(15)	57	(15)
	2012	111	(11)	38	(25)	58	(16)
Assignment location, region*	South	633	(64)	118	(78)	223	(61)
	West	149	(15)	22	(14)	51	(14)
	Non-US	116	(12)	7	(5)	58	(16)
	Midwest	46	(5)	4	(3)	23	(6)
	Northeast	13	(1)	0	(0)	5	(1)
	Territory	3	(0)	0	(0)	1	(0)
CD3 CD4, absolute (cells/μl)	249 or less	91	(9)	10	(7)	34	(9)
	250–500	404	(41)	74	(49)	151	(41)
	501–1979	390	(39)	63	(42)	172	(47)
Viral load	<10k	228	(23)	33	(22)	100	(27)
	10-99k	313	(31)	74	(49)	150	(41)
	>100k	115	(12)	23	(15)	51	(14)
Any STI	Yes	136/848	(14)	34/148	(22)	54/352	(15)
Hepatitis B, acute	Yes	5/719	(0)	1/135	(1)	1/303	(0)
Hepatitis C, chronic	Yes	6/723	(1)	1/139	(1)	1/299	(0)
Syphilis	Yes	85/591	(8)	19/116	(12)	38/243	(11)
*Neisseria gonorrhea*	Yes	22/435	(2)	7/108	(4)	7/203	(2)
*Chlamydia trachomatis*	Yes	36/435	(4)	15/107	(9)	12/205	(3)
Last HIV test (days)	<180	143	(14)	26	(17)	46	(12)
	181–365	234	(23)	35	(23)	101	(27)
	366–730	393	(39)	77	(51)	160	(44)
	>730	214	(21)	13	(9)	58	(16)
ART naïve^†^	Yes	400/443	(40)	127/132	(84)	273/311	(74)
Transmitted drug resistance	Yes	155/518	(16)	54/151	(36)	101/367	(27)

Notes: n (%) unless otherwise indicated; missing values are not shown.

*States were categorized into region using U.S. Census Bureau definitions; the Southern U.S. includes Alabama, Arkansas, Delaware, Florida, Georgia, Louisiana, Kentucky, Maryland, Mississippi, Oklahoma, North Carolina, South Carolina, Tennessee, Texas, Virginia, West Virginia, and the District of Columbia

^†^Among those with documented evidence of antiretroviral prescriptions (n = 441), for 9% who had no sequence on file before ART was initiated, the next available sequence after ART initiation was used in analysis.

Among HIV-infected Soldiers, 518 (52%) had a partial *pol* sequence available for analysis. A majority (79%) of sequences were generated after 2006 and a median 50 days (IQR 27.0–257.0) post-diagnosis. Subtyping of the *pol* fragment indicated HIV-1 subtype B infection predominated among HIV-infected Soldiers (94%); non-B subtypes identified were A (1%), C (1%), G (1%) and inter-subtype recombinants (3%: AB, AD, BC, and CRF02/B– 0.2% each, CRF01–0.6%, CRF02–1.2%). Among antiretroviral therapy (ART)-naïve Soldiers with subtype B infection (n = 374), resistance to non-nucleoside reverse transcriptase inhibitor drugs was most common (22%) followed by resistance to nucleoside reverse transcriptase inhibitor (7%) and major protease inhibitors (3%). We classified 29% (47 pairs, 16 non-pairs) of sequences as being clustered; 96% were subtype B infections and remaining subtypes were A (1.3%), C (1.3%), and G (1.3%). Clusters were composed of a median of 2 members (IQR 2.0–3.0, mean 2.4, range 2.0–6.0).

### Characteristics of membership in transmission clusters

In univariate analysis, HIV-infected Soldiers with membership in a cluster were slightly younger (mean 28 vs 29), more frequently of black, non-Hispanic racial and ethnic origin (68% vs 57%), differed in calendar year and duty location of diagnosis, were more likely to have a STI co-infection (22% vs.15%), less frequently initiated ART (84% vs 74%), and differed in HIV-1 TDR resistance (36% vs 27%) compared to Soldiers who were not part of a transmission pair/cluster (p≤0.05, Table 1). Among those who were clustered, 98% were linked to other male Soldiers; of the 2% who were not, 2 female Soldiers were linked to each other and were of black or other race, and 1 female was linked to a male Soldier (both were of black race). Of black, non-Hispanic Soldiers in clusters, 69% were linked to other black Soldiers and of white, non-Hispanic Soldiers, 26% were linked to other white Soldiers while 59% were linked to black Soldiers. Among 5 Soldiers who were ART experienced, and who were all members of transmission pairs, 3 had HIV-1 sequences with a TDR mutation; the other members of the pairs were ART naïve with HIV-1 sequences having no TDR mutations. Transmitted drug mutations were found more commonly among clusters with a membership size of 3 or more (member n = 32, 59%) compared to transmission pairs (member n = 22, 41%).

In multivariate analysis, after adjustment for other significant characteristics (p<0.10), only two factors were associated independently with cluster membership (Table 2). Soldiers assigned to a southern region at diagnosis were almost twice as likely to be part of a transmission cluster compared with Soldiers assigned elsewhere [adjusted odds ratio (AOR) 1.94, 95% confidence interval (95% C.I.) 1.19–3.18]. The odds of having membership in a transmission network increased 12% for each calendar year of HIV diagnosis (AOR 1.12, 95% C.I. 1.03–1.23).

**Table 2:** Characteristics of 518 newly diagnosed HIV-infected Soldiers associated with transmission clustering, 2001–2012.

Characteristic at diagnosis	Value	Unadjusted OR	95% C.I.	P-value	Adjusted OR*	95% C.I.	P-value
Age (years)	One year	0.97	(0.95–0.99)	0.040	0.98	(0.94–1.01)	0.143
Race, ethnicity	Black, non-Hispanic/Other vs White, non-Hispanic	1.67	(1.04–2.69)	0.034	1.60	(0.91–2.81)	0.103
Marital status	Single vs non-single	0.89	(0.60–1.30)	0.538	-	-	-
Education	High school or less vs Some college or more	1.32	(0.82–2.13)	0.258	-	-	-
Grade	E1-E4 vs Officer	1.63	(0.79–3.33)	0.184	-	-	-
	E5-E9 vs Officer	1.80	(0.87–3.70)	0.112	-	-	-
Length in service (years)	One year	0.98	(0.95–1.01)	0.225	-	-	-
Occupation category	Combat vs Other	1.50	(0.91–2.47)	0.112	-	-	-
	Health Care vs Other	0.74	(0.40–1.37)	0.339	-	-	-
Calendar year	One year	1.13	(1.06–1.22)	0.001	1.12	(1.03–1.23)	0.013
Assignment location (region)	South vs Other	2.21	(1.42–3.44)	0.000	1.94	(1.19–3.18)	0.008
CD3 CD4 (cells/μl)	100 cells	0.97	(0.89–1.06)	0.478	-	-	-
Viral load (copies/mL)	<10,000 vs. >100,000	0.73	(0.39–1.37)	0.331	-	-	-
	10,000–99,999 vs >100,000	1.09	(0.62–1.93)	0.756	-	-	-
Any STI	Yes vs No	1.65	(1.02–2.66)	0.042	1.45	(0.84–2.51)	0.177
Last negative test (months)	One month	0.98	(0.96–1.00)	0.044	0.99	(0.97–1.00)	0.145
ART naïve	Yes vs No	3.53	(1.36–9.19)	0.010	2.82	(0.89–8.92)	0.077
Transmitted drug resistance	Yes vs No	1.47	(0.98–2.20)	0.060	1.55	(0.97–2.48)	0.069

*Variables were adjusted for characteristics of significance (p-value<0.10) in univariate analysis.

## Discussion

We evaluated demographic and clinical characteristics of 995 active duty Soldiers newly diagnosed with HIV from 2001 to 2012 and explored characteristics associated with membership in transmission clusters among 518 HIV-infected Soldiers with partial *pol* sequences. More than a third had an HIV diagnosis within one year of the last negative HIV test and a significant proportion of Soldiers had received HIV specialty care within 3 months of HIV diagnosis. Subtype B infection predominated (94%) similar to HIV-1 infections identified by the NHSS in the U.S. (93.3%).[12] Among 29% of Soldiers who were members of transmission clusters, two characteristics were associated independently with clustering: assignment to a southern U.S. location at diagnosis and year of diagnosis.

Several factors that increase HIV acquisition and transmission may explain the association between duty assignment and cluster membership. U.S. national surveillance data from 2008 to 2012 indicated that southern states had the highest diagnosis rate of HIV, especially among nine states (Alabama, Florida, Georgia, Louisiana, Mississippi, North and South Carolina, Tennessee and Texas) referred to as the Deep South, and the highest HIV-related death rate even after adjustment for factors at diagnosis such as age, race, sex, transmission category and residence indicating that factors other than these were influential in HIV outcomes.[13] Influential factors may include low state population density of lesbian, gay, bisexual, and transgender individuals, income inequality, high syphilis rates, insufficient or delayed access to health care including testing for HIV, and receiving HIV care (only 30% with HIV were virally suppressed in 2011), lack of awareness of HIV infection status, poor overall health, and HIV-related stigma.[14–16] Although the U.S. HIV diagnosis rate decreased from 2005 to 2014, the rate increased among black/African American MSM,[17] especially among black MSM in the South, who accounted for more than half of HIV diagnoses in 2014.[18] Assignment in the South may have increased the likelihood of HIV acquisition especially among black/African American Soldiers. Recent Army HIV behavioral surveillance data indicate that a majority of HIV-infected Soldiers were black/African American and MSM who believed they acquired HIV in southern U.S. states.[4] Among MSM, 64% reported having civilian partners and 36%, service member partners. Black/African American HIV-infected men in the U.S. were more likely to be linked to partners of the same race than white HIV-infected men (81% versus 62%, respectively).[3]

Increasing calendar year of HIV diagnosis was associated independently with cluster membership. Although it is likely surveillance or sampling bias may have increased the likelihood of detecting transmission networks as more sequence data accumulated over time due to increased antiretroviral testing from compliance with national guidelines for earlier initiation of ART,[19] characteristics of the national HIV epidemic may also explain the association. National HIV data indicated a predicted increase in incidence in the U.S. since 2001 with an estimated 50,000 infections occurring nationally each year.[20] Contributors to forward transmission included lack of awareness of HIV infection status in up to one-fifth of those infected and less than half of the HIV-infected population on antiretroviral therapy in 2011 with the lowest proportion of viral suppression among those aged 18–34 years.[14] It is likely that the same contributory factors for transmission in the national HIV epidemic are impacting the military HIV epidemic. Recent interview data from HIV-infected Soldiers indicate epidemiological linkage with members of the national civilian population.[4] Army regulations mandate force-wide biennial HIV testing.[21] However, our analysis indicates more than a third of HIV-infected Soldiers (and more than half of Soldiers who were clustered) could have benefited from annual, rather than biennial, testing and subsequent earlier linkage to specialty care due to earlier diagnosis. CDC’s 2006 guidelines recommend at least annual testing among high-risk persons.[22] Furthermore, studies indicate that testing high risk persons (populations with ≥1.0% incidence) every three months is cost-effective.[23, 24] One such study which evaluated testing intervals using fourth-generation assays among MSM found even more frequent testing such as quarterly or biannual testing was cost effective compared to annual testing.[23]

These analyses had limitations. The transmission clusters that were observed do not indicate the direction of transmission or actual transmission events since un-sampled individuals may be involved. Furthermore, the strict criteria that we used to define clustering as well as undersampling of the infected population may have led to an underestimation of the magnitude of clustering among HIV-infected Soldiers.

In conclusion, despite unavailability of sequences for a large proportion of HIV-infected Soldiers and use of strict criteria for identifying clusters, we effectively utilized routinely available clinical sequence data in HIV surveillance to identify factors associated with cluster membership. These findings corroborate recent behavioral surveillance data and indicate that further study of transmission networks using military enterprise-wide sequence coverage and transmission category data are warranted as well as a renewed cost analyses of targeted HIV prevention efforts. An increased frequency of current force testing may be justified, especially among Soldiers assigned to duty in installations with high local HIV prevalence such as southern U.S. states.

## Supporting information

S1 Fig Phylogenetic tree of assignment of viral subtypes and significant transmission clusters. A phylogenetic analysis of viral subtypes, with respect to 40 reference sequences, and significant transmission clusters using the maximum likelihood method in MEGA6. The subtypes are denoted by purple, blue, and green arcs. The transmission clusters with bootstrap values of ≥95% and genetic distances of ≤1.5% are illustrated as red dots.
(JPG)

S2 Fig The distribution of pair-wise distances between clustered sequences compared to all sequences. The histogram depicts the distribution of pair-wise distances between clustered sequences compared to all sequences.
(JPG)

S3 Fig Comparison of genetic distances generated using two pair-wise distance models. A comparison of genetic distances generated using two pair-wise distance models, Kimura’s two parameter (1980) and Tamura-Nei (1993) (Pearson product-moment correlation coefficient = 0.999)
(JPG)

S1 Table List of forty reference sequences that were used for subtyping in phylogenetic analysis. Forty references consisting of pure HIV-1 subtype and main circulating recombinant forms.
(DOCX)
