# Supplementary figures and images for: Characteristics of HIV-infected U.S. Army soldiers linked in molecular transmission clusters, 2001-2012
Source: PLoS One. 2017 Jul 31;12(7):e0182376. doi: 10.1371/journal.pone.0182376 (PMC5536263; doi:10.1371/journal.pone.0182376)

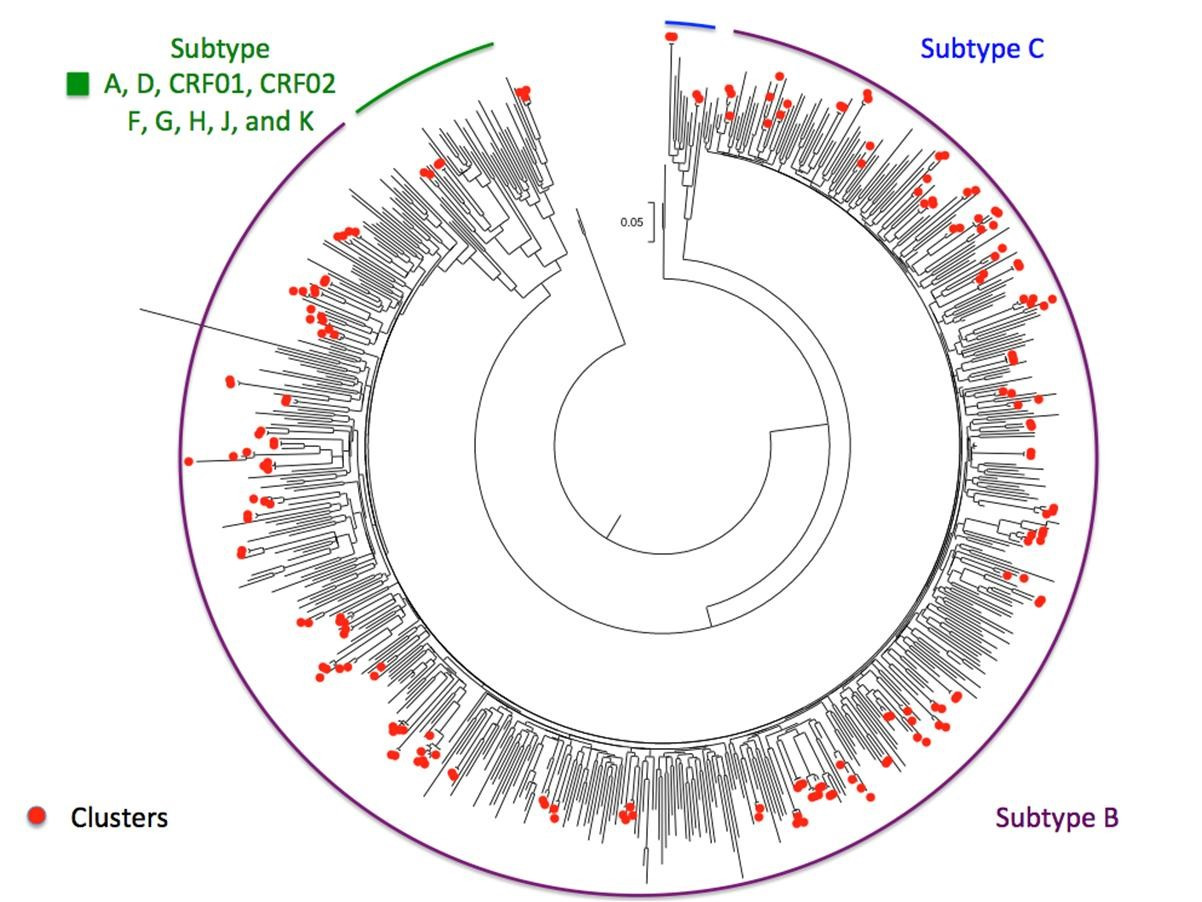

Supplement: S1 Fig — A phylogenetic analysis of viral subtypes, with respect to 40 reference sequences, and significant transmission clusters using the maximum likelihood method in MEGA6. The subtypes are denoted by purple, blue, and green arcs. The transmission clusters with bootstrap values of ≥95% and genetic distances of ≤1.5% are illustrated as red dots. (JPG) [file pone.0182376.s001.jpg]

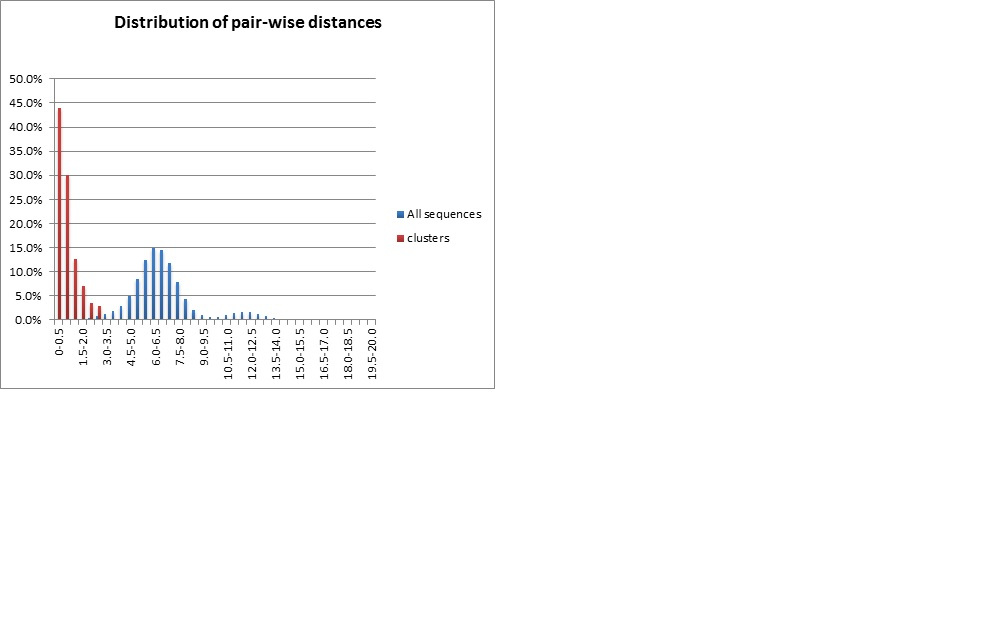

Supplement: S2 Fig — The histogram depicts the distribution of pair-wise distances between clustered sequences compared to all sequences. (JPG) [file pone.0182376.s002.jpg]

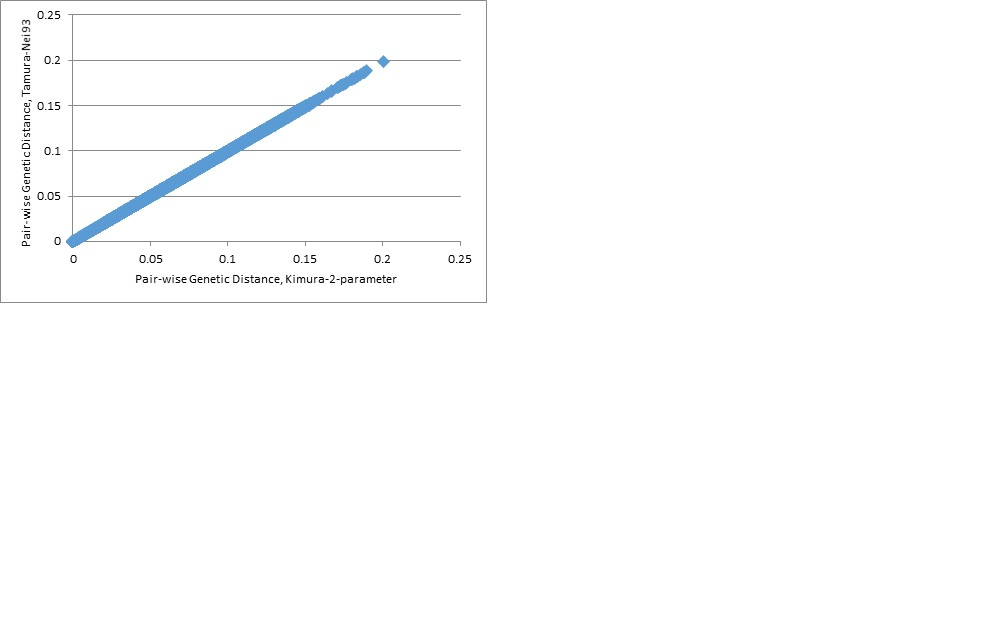

Supplement: S3 Fig — A comparison of genetic distances generated using two pair-wise distance models, Kimura’s two parameter (1980) and Tamura-Nei (1993) (Pearson product-moment correlation coefficient = 0.999) (JPG) [file pone.0182376.s003.jpg]
