# Supplementary material for: Characteristics of HIV-infected U.S. Army soldiers linked in molecular transmission clusters, 2001-2012
Source: PLoS One. 2017 Jul 31;12(7):e0182376. doi: 10.1371/journal.pone.0182376 (PMC5536263; doi:10.1371/journal.pone.0182376)
Supplement: S1 Table — Forty references consisting of pure HIV-1 subtype and main circulating recombinant forms. (DOCX) [file pone.0182376.s004.docx]

SUPPLEMENTAL DATA

Supplemental Figure 1

Phylogenetic tree of assignment of viral subtypes, with respect to 40 reference sequences, and significant transmission clusters. The subtypes are denoted by purple, blue, and green arcs. The transmission clusters with bootstrap values of ≥95% and genetic distances of ≤1.5% are illustrated as red dots.


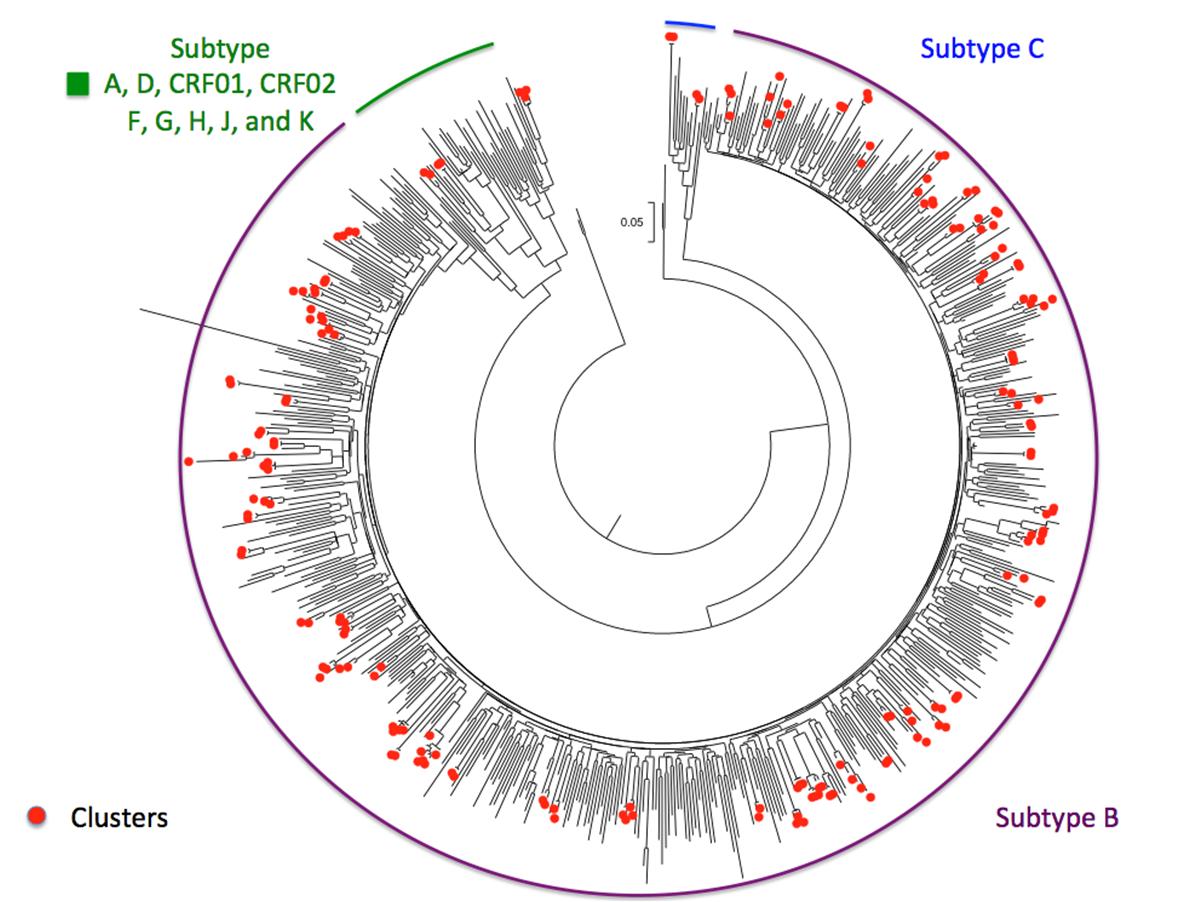


Supplemental Table 1

List of forty reference sequences that were used for subtyping in phylogenetic analysis

| **Accession ID** | **HIV Subtype** |
| --- | --- |
| AF061642 | G |
| AF286237 | A2 |
| AF286238 | A2 |
| AF457069 | A1 |
| AF457079 | A1 |
| AF457090 | D |
| AF484477 | D |
| AJ249238 | F1 |
| AY173952 | B |
| AY173955 | B |
| AY253304 | C |
| AY304496 | D |
| AY371127 | AG |
| AY371128 | AG |
| AY444801 | C |
| AY444805 | AE |
| FJ388925 | A1 |
| AY713408 | B |
| AY713411 | B |
| AY713414 | C |
| AY713417 | C |
| AY713419 | AE |
| AY713422 | AE |
| AY713425 | AE |
| AY713426 | AE |
| GQ477441 | AE |
| JX140673 | F2 |
| K03455 | B |
| U88826 | G |
| GU201513 | AG |
| AF082394 | J |
| AF082395 | J |
| AJ249235 | K |
| AJ249239 | K |
| FJ670530 | G |
| JN248584 | G |
| JX140646 | AG |
| U71182 | B |
| DQ354114 | B |
| GU201516 | A2 |

Supplemental Figure 2

The histogram depicts the distribution of pair-wise distances between clustered sequences compared to all sequences.

Supplemental Figure 3

A comparison of genetic distances generated using two pair-wise distance models, Kimura’s two parameter (1980) and Tamura-Nei (1993) (Pearson product-moment correlation coefficient=0.999)
